# Can the cross-education of strength attenuate the impact of detraining after a period of strength training? A quasi-randomized trial

**DOI:** 10.1007/s00421-024-05509-z

**Published:** 2024-05-29

**Authors:** Grant S. Rowe, Anthony J. Blazevich, Janet L. Taylor, Timothy Pulverenti, G. Gregory Haff

**Affiliations:** 1 School of Psychology, College of Health and Education, Murdoch University, 90 South Street, Murdoch, Perth, 6150 Australia; 2 Discipline of Exercise and Sports Science, School of Medical and Health Sciences, Edith Cowan University, Joondalup, Perth, Australia; 3 Department of Anaesthesiology, Montefiore Medical Center, New York, USA; 4 Directorate of Sport, Exercise, and Physiotherapy, University of Salford, Greater Manchester, UK; 5 Strength and Power Research Group, School of Medical and Health Sciences, Edith Cowan University, Joondalup, Perth, Australia

**Keywords:** Unilateral, Cross-education of strength, Muscle strength, Neural, Muscle size

## Abstract

**Purpose:**

Unilateral strength training may attenuate the decline in muscle strength and size in homologous, contralateral muscles. This study aimed to determine whether the cross-education of strength could specifically attenuate the effects of detraining immediately after a short (prehabilitation-type) period of strength training.

**Methods:**

Twenty-six strength-trained participants were assigned to either four weeks of unilateral strength training of the stronger arm (UNI) or detraining (Detrain). Motor evoked potential (MEP) and cortical silent period (cSP) responses, muscle cross-sectional area (CSA_Flexor_; peripheral quantitative computed tomography) and maximal strength, rate of force development (RFD) and muscle activation (EMG) were examined in both elbow flexors before and after the intervention period.

**Results:**

In UNI, one-repetition maximum (1-RM) strength improved in both the trained (∆ = 2.0 ± 0.9 kg) and non-trained (∆ = 0.8 ± 0.9 kg) arms despite cessation of training of the weaker arm, whereas 1-RM strength was unchanged in Detrain. Maximal voluntary isometric contraction, isokinetic peak torque, and RFD did not change in either group. No neural changes were detected in UNI, but cSP increased in Detrain (∆ = 0.010 ± 0.015 s). CSA_Flexor_ increased in the trained arm (∆ = 51 ± 43 mm^2^) but decreased in the non-trained arm (∆ = -53 ± 50 mm^2^) in UNI. CSA_Flexor_ decreased in both arms in Detrain and at a similar rate to the non-trained arm in UNI.

**Conclusion:**

UNI attenuated the effects of detraining in the weaker arm as shown by the improvement in 1-RM strength. However, the cross-education of strength did not attenuate the decline in muscle size in the contralateral arm.

**Supplementary Information:**

The online version contains supplementary material available at 10.1007/s00421-024-05509-z.

## Introduction

It is well established that unilateral or single muscle group strength training provides a stimulus to the homologous muscle(s) of the contralateral limb (Carroll et al. 2006). The repeated generation of this stimulus enables the homologous muscles of the contralateral limb to become stronger without exercising (Carr et al. 2019), a phenomenon referred to as the *cross-education of strength* (Manca et al. 2021; Lee and Carroll 2007). This effect has important practical implications in rehabilitation settings, especially for individuals during a period of limb immobilization due to musculoskeletal injury or post-surgery recovery (Hendy, Spittle, Kidgell 2012). A prominent adverse effect of immobilization is a loss of muscular strength and size in the immobilized limb (Farthing, Krentz, Magnus 2009), prolonging the recovery time needed to restore mechanical function. Previous research has shown that unilateral strength training during upper limb immobilization enables the retention of maximal strength, muscle thickness, and corticospinal excitability in the homologous, contralateral muscle (Pearce et al. 2013; Farthing et al. 2011). However, little is known about whether unilateral strength training can help the retention of muscular strength, muscle size, or corticospinal properties in a homologous, contralateral muscle during a period of detraining in a strength-trained muscle.

Detraining occurs when an individual either ceases training or reduces the training stimulus below a critical threshold, causing maladaptation and a loss in functional capacity (Mujika and Padilla 2008). These maladaptive responses can result in reductions in maximal strength in as little as 3–5 weeks (McMaster et al. 2013). Similarly, the rate of force development (RFD) can decline within three weeks of training cessation (Kobayashi et al. 2013). Neural factors have been suggested to be the primary cause of these short-term detraining effects (Häkkinen and Komi 1983). For instance, significant decreases in EMG activity and voluntary activation were found to accompany decreases in maximal voluntary isometric contraction (MVIC) torque following four weeks of detraining (Gondin et al. 2006). Although most detraining studies investigate these maladaptations by enforcing total-body training cessation, there are situations in which trained individuals are advised not to exercise one of their limbs, e.g., acute or overuse injuries, where limb-specific decay of muscle activation, size, and strength are likely to occur. As the contralateral limb remains able to perform high-intensity muscle contractions in this situation, clinicians and practitioners may prescribe unilateral training to counter the adverse effects of detraining in the exercise-restricted limb (Hendy et al. 2012; Farthing, Krentz et al. 2009). However, the cross-education of strength may be relatively ineffective in trained individuals as the proposed cortical adaptations (Ruddy and Carson 2013; Lee et al. 2010) underlying the response may already be induced. In support of this theory, a recent study has reported that unilateral leg extensor training prescribed at increasing weekly loads (70–85% of one-repetition maximum [1-RM] for 4 weeks) was unable to maintain muscle strength and power in the contralateral leg extensors of recreationally engaged older women (> 60 years) (de Souza Teixeira et al. 2023). With numerous studies demonstrating greater cross-education benefits training with heavier loads (Pelet and Orsatti 2021; Voskuil et al. 2023), the question of whether unilateral strength training prescribed at higher intensities is effective in the reduction of detraining effects is still open. This finding is important for athletes and otherwise-active populations who have become injured as well as for clinical practitioners who use exercise ‘prehabilitation’ to improve the recovery rate of their patients from a pre-planned surgery.

Therefore, the question of whether strength loss is significant after cessation of a short-term (prehabilitation-type) period of strength training (performed in our previous study: Rowe et al. 2023) and whether any potential loss is reversible with high-intensity training of the other arm remains to be investigated. It was hypothesized that the cross-education of strength would not be effective in attenuating the loss in muscle activation and size in a previously trained muscle and, therefore, decreases in muscle strength in the contralateral limb would occur despite unilateral strength training.

## Methods

### Participants

Twenty-six participants (age: 25 ± 5 years; height: 1.7 ± 0.9 m; body mass: 68.9 ± 13.9 kg) completed four weeks of elbow flexion strength training of both arms immediately before the present tests were commenced (described in our previous study: Rowe et al. 2023). Participants were eligible to take part in the study if they had not experienced an upper body musculoskeletal injury in the last 3 years, were not taking medications that might affect neuromuscular and strength adaptation, and had no contraindications to transcranial magnetic stimulation (TMS) according to a TMS safety checklist (Rossi et al. 2011). Participants were instructed to abstain from any high-intensity upper body activities and to maintain their current diet until the conclusion of the study. They were also instructed to abstain from caffeinated or alcoholic drinks for 6 and 12 h before each testing session, respectively. The University Human Research Ethics Committee approved the study (#16,826), and all participants provided written informed consent before commencement.

### Experimental approach

**Fig. 1:**
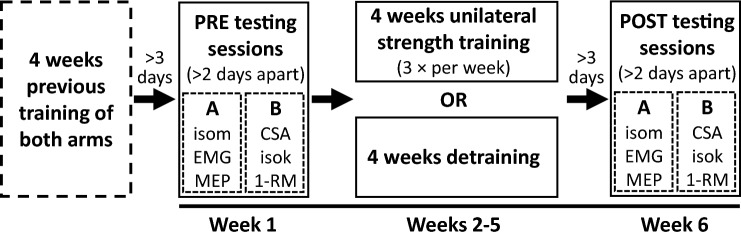
Experimental approach. The study compared 4 weeks of unilateral strength training or detraining (no training) on strength-trained muscles. Multiple parameters were assessed for both arms before and after the 4-week intervention. A = pre- and post-training testing session A, B = pre- and post-training testing session B, isom = isometric; EMG = electromyography; MEP = motor evoked potential; CSA = cross-sectional area; isok = isokinetic; 1-RM = 1-repetition maximum; previous training = participants completed 4 weeks of strength training in an earlier study (Rowe et al. 2023)

Participants were assigned to one of the two experimental groups: unilateral strength training (UNI) or detraining (Detrain). Participants were quasi-randomized into the groups based on their 1-RM strength and sex, with the researchers determining suitable individuals who matched those already randomly allocated in the groups (UNI; males: *n* = 7, 13.2 ± 1.8 kg, females: *n* = 6, 8.7 ± 1.2 kg; Detrain; males: *n* = 6, 15.8 ± 2.9 kg, females: *n* = 7, 8.4 ± 0.9 kg). UNI attended 12 training sessions across the 4-week training period. Both groups participated in two testing sessions at the beginning of the study and two testing sessions at the end. The pre-training testing sessions were completed at least three (PRE_A_) and six (PRE_B_) days after the cessation of the previous training (Rowe et al. 2023) and at least two days apart. In PRE_A_, isometric strength, muscle activation (via EMG), and TMS responses were examined, while muscle cross-sectional area (CSA) and isokinetic and isoinertial strength were assessed in PRE_B_. In UNI, participants continued free-weight biceps curl training of only the stronger arm for 4 weeks, while Detrain ceased training of both arms. UNI performed post-training testing sessions at least three (POST_A_) and six (POST_B_) days after the cessation of training and at least two days apart. Over the same week, Detrain performed their post-detraining testing sessions (POST_A_ and POST_B_) at least two days apart. Figure 1 shows the experimental approach to the study.

### Experimental procedures

#### Pre- and post-training testing session A

Isometric strength, muscle activation (via EMG), M-wave, and TMS tests were conducted on both arms at PRE_A_ and POST_A_. Arms were tested in a random order with all testing completed first on one arm and then the other. During these sessions, the participant was seated on an isokinetic dynamometer chair (Biodex System 4 Pro, Shirley, NY) with the elbow supported at 90° and aligned with the dynamometer’s axis of rotation. Surface electrodes (Ag–AgCl) were placed in a muscle–tendon montage (mid-belly of the biceps brachii and its distal tendon), with the reference electrode positioned over the humeral lateral epicondyle (Blue Sensor N-00-S, 28 mm^2^, Ambu, Ballerup, Denmark). Participants were instructed to pull up against the dynamometer at the wrist, instead of holding the elbow flexor attachment, to limit grip-related effects impacting TMS measurement (Hasegaw et al. 2001). An inelastic cord was used to fix the wrist to the handle. MVIC torque and RFD were examined with the participant pulling upwards *“as fast and then as hard as possible”*. Testing consisted of at least three 3-s MVICs with 1 min of passive rest between trials. Participants performed more than three trials if peak torque of the third and later trials were > 5% greater than the earlier trials. This procedure ensured that peak torque reached a plateau before MVIC testing was concluded. The participant then performed three additional trials with the instruction to contract *“as fast as possible”*, although their peak torque had to reach at least 80% of MVIC torque (Folland, Buckthorpe, Hannah 2014). These additional trials were conducted so that maximum RFD was also detected.

Following the isometric strength testing, cathode and anode stimulation electrodes (White Sensor 4560M, 79 mm^2^, Ambu, Ballerup, Denmark) were placed on the participant’s supraclavicular fossa (Erb’s point) and the acromion process, respectively. The maximal biceps brachii M-wave (M_max_) was evoked with 200-µs duration electrical stimuli delivered using a constant-current stimulator (DS7AH, Digitimer, Welwyn Garden City, UK). Upon reaching the lowest intensity that generated M_max_, stimulation intensity was increased by 20%, and the mean M-wave amplitude (mV) recorded from two stimuli was used in analyses.

Finally, TMS was delivered to the upper arm motor area of the contralateral cerebral hemisphere using a Magstim 200^2^ stimulator (Magstim Co, Dyfed, UK) and a 90 mm figure-of-8 coil. After locating the hotspot for biceps brachii, the active motor threshold (AMT) was determined with the participant contracting to a target of 5% of peak muscle activity (EMG_peak_). AMT was defined as the minimum stimulator output (SO) that generated a motor evoked potential (MEP) of 2 SD above the background EMG. Maximum-likelihood parameter estimation by sequential testing (PEST) was used to determine AMT (Awiszus and Borckardt 2011). Subsequently, 10 single-pulse stimuli (~ 10 s) were delivered during 5% EMG_peak_ contraction at block-randomized stimulator intensities of 120% and 150% of AMT.

#### Pre- and post-training testing session B

Within sessions PRE_B_ and POST_B_, CSA as well as isokinetic and isoinertial strength of the elbow flexors were examined in both arms. First, whole-upper arm CSA was measured at a single location using peripheral quantitative computed tomography (pQCT; XCT-3000; Stratec Medizintechnik, Pforzheim, Germany). The participant lay supine with a single arm placed in an abducted position through the pQCT gantry. A single-slice CT scan was performed at 33% of the humeral segment length (reference line positioned on the proximal endplate of the radius), with the segment length estimated as 0.186 × standing height (measured at PRE). From these images, elbow flexor CSA (CSA_Flexor_) was measured, as described in detail in Data Analysis (below).

Subsequently, the participant was tested for maximal unilateral isokinetic elbow flexion strength of each arm on the isokinetic dynamometer (as above) at angular velocities of 210°·s^−1^ and then 20°·s^−1^. These angular velocities were tested as they reflect fast and slow speeds with substantial and clear differences between them. The participant held the elbow attachment handle and positioned their elbow underneath their shoulder while lightly contacting the chair backrest. The elbow position was aligned with the dynamometer’s axis of rotation. Starting with the elbow slightly bent (~ 10°; vertical lever arm = 0°), strength was assessed through a 90° range of motion. Three single concentric elbow flexion trials separated by 1 min of passive rest for each velocity were completed with the highest peak torque (Nm) for each velocity used for data analysis.

Finally, unilateral 1-RM strength of each arm was tested on an adjustable preacher curl bench (Sorinex, South Carolina, USA) to assess maximal isoinertial elbow flexion strength. Starting from elbow flexion, the participant lowered the dumbbell to near-maximal elbow extension before returning to the starting position. Two min after each successful lift, a heavier load was attempted until a repetition could not be completed. The arm that was recorded as ‘stronger’ before the commencement of the previous training (see previous study: Rowe et al. 2023) was noted as stronger for this study.

### Data analysis

A computer running LabChart software (version 8.1.9, ADInstruments, New South Wales, Australia) and a 16-bit analog-to-digital converter sampling at 2,000 Hz (PowerLab 16/35, ADInstruments, New South Wales, Australia) were used to collect torque and EMG signals during isometric strength testing. The torque signals were smoothed with an 18-Hz low-pass filter (linear-phase finite impulse response [FIR] filter), while EMG signals were filtered with a 20–500 Hz band-pass FIR filter (gain = 1,000, transition width = 4 Hz, input impedance = 200 MΩ, common-mode rejection ratio ≥ 85 dB at 1–60 Hz). The MVIC trial that generated the highest peak torque (Nm) and the trial, either MVIC or 80% of MVIC, that generated the greatest RFD (torque onset to 50 ms; Nm·s^−1^) were used in subsequent analyses. RFD was calculated as the slope of the torque–time curve from torque onset to 50 ms (RFD_50_) as well as from 100 to 200 ms (RFD_100-200_) after torque onset. Subsequently, RFD was normalized (%) to each participant’s MVIC torque (i.e., RFD_50/MVIC_ and RFD_100-200/MVIC_). The technique used to determine force onset (as well as EMG onset) has been defined elsewhere (Tillin et al. 2010).

The root mean square (RMS) amplitudes of EMG from EMG onset to 40 ms (EMG_40_) and 100 ms (EMG_100_) in the best RFD trial as well as the RMS EMG within a 500-ms window before peak torque from the best MVIC trial (EMG_peak_) were calculated as measures of muscle activation. All values were then normalized to M_max_ (%M_max_). Peak-to-peak MEP amplitudes were measured and averaged for responses at 120% and 150% of AMT and subsequently normalized to M_max_ (%M_max_). Additionally, the duration of the cSP was quantified as the time (s) from stimulus artifact to the reoccurrence of ongoing voluntary EMG (50% of pre-stimulus EMG) and averaged for each stimulus intensity. Pre-stimulus EMG was calculated as the mean rectified EMG over 100 ms before stimulation (%M_max_).

CSA_Flexor_ (mm^2^) was calculated from whole-upper arm images by outlining the muscle group with the polygon tool in ImageJ software (National Institutes of Health, Maryland, USA) (Schneider et al. 2012). An example of the outlining technique used with the pQCT images to assess CSA_Flexor_ can be found elsewhere (Rowe et al. 2023).

The cross-education of strength changes (1-RM strength, isokinetic peak torque at 20°·s^−1^ and 210°·s^−1^ and MVIC torque) were determined using a formula originally reported by Carroll and colleagues (Carroll et al. 2006). It calculates the difference between groups in the mean strength change in the weaker arm (i.e., non-trained arm in UNI) following the intervention period:

$$\left[ {\frac{{UNI_{post} {-} UNI_{pre} }}{{UNI_{pre} }} {-} \frac{{Detrain_{post} {-} Detrain_{pre} }}{{Detrain_{pre} }}} \right] \times 100,$$

where UNI_pre_ and UNI_post_ refer to mean strengths of the non-trained arm in UNI, and Detrain_pre_ and Detrain_post_ refer to mean strengths of the weaker arm in Detrain, before and after the intervention period.

### Unilateral strength training

In UNI, the participants completed four weeks of unilateral biceps preacher curl training three times per week with the stronger arm. Two warm-up sets of unilateral dumbbell preacher curls were performed for 12 and 8 repetitions at 60% and 75% of the participants pre-training 1-RM load, respectively. The subsequent training was comprised of 4 sets of 3–5 repetitions with a load equivalent to 90% of their pre-training 1-RM. The same lifting technique used during the 1-RM test was used during training; however, a 3-min passive rest was prescribed between sets. A metronome was used to provide timing to the participant, with 3 s and 2 s devoted to eccentric and concentric phases, respectively. The load was continually adjusted to ensure that the participant was unable to complete more lifts than the prescribed repetition range. A set was deemed complete when the load could not be lifted, or two consecutive repetitions were performed with a concentric phase exceeding the 2-s limit.

### Statistical analysis

Normality of data was confirmed using Shapiro–Wilk testing and quantile–quantile (Q–Q) plots and data were transformed (rank-based inverse transformation) in the event of non-normality (Templeton 2011). Data parameters that required transformation were 1-RM strength, isokinetic peak torque at 20°·s^−1^, RFD, EMG, and MEP amplitudes. Three-way (arm [stronger versus weaker], time [PRE versus POST] and group [UNI versus Detrain]) repeated-measures MANOVAs (RFD, early EMG, and pre-stimulus EMG) and ANOVAs (all other dependent variables) were used to compare the responses to strength training and detraining. Pairwise comparisons (with Holm–Bonferroni Sequential adjustment) were reported when significant interaction (group × arm, group × time, arm × time, group × arm × time) effects were detected. Additionally, a one-way repeated-measures ANOVA was used to compare the differences between cross-education of strength scores. Effect sizes are reported using Hedges *g* and interpreted as trivial (g < 0.2), small (0.2 ≤ g < 0.5), moderate (0.5 ≤ g < 0.8), or large (g ≥ 0.8). Links between specific dependent variables (RFD_MVIC_ vs. early EMG change scores; MVIC vs. EMG_peak_ change scores; 1-RM strength vs. CSA_Flexor_ change scores) were also investigated using Pearson’s product-moment correlations (*r*) with 95% confidence intervals (CI) (bias-corrected accelerated bootstrapping). Significant relationships were reported when the 95% CI did not cross 0.00. The alpha level was set at 0.05 for all other analyses, and data are presented as mean ± SD. Statistical computations were performed using a statistical analysis program (SPSS, Version 25.0; Chicago, Illinois, United States).

## Results

### Training loads in UNI

The loads lifted (kg) and repetitions completed with the stronger arm in the unilateral training group are summarized in Table 1. Across the training period, the loads used in training increased from 11.1 ± 2.2 kg to 12.6 ± 2.3 kg.

**Table 1:** The loads lifted (kg) and repetitions completed during unilateral strength training of the stronger arm in the UNI group

Mean load per set (kg)				Repetitions per set (**n**)			
Sessions 1–4	Sessions 5–8	Sessions 9–12	4-week average	Set 1	Set 2	Set 3	Set 4
11.1 (2.2)	11.8 (2.3)	12.6 (2.3)	11.8 (2.3)	4.7 (0.7)	4.3 (0.6)	4.1 (0.5)	4.2 (0.4)

Mean (SD) are shown

### 1-RM strength

**Fig. 2:**
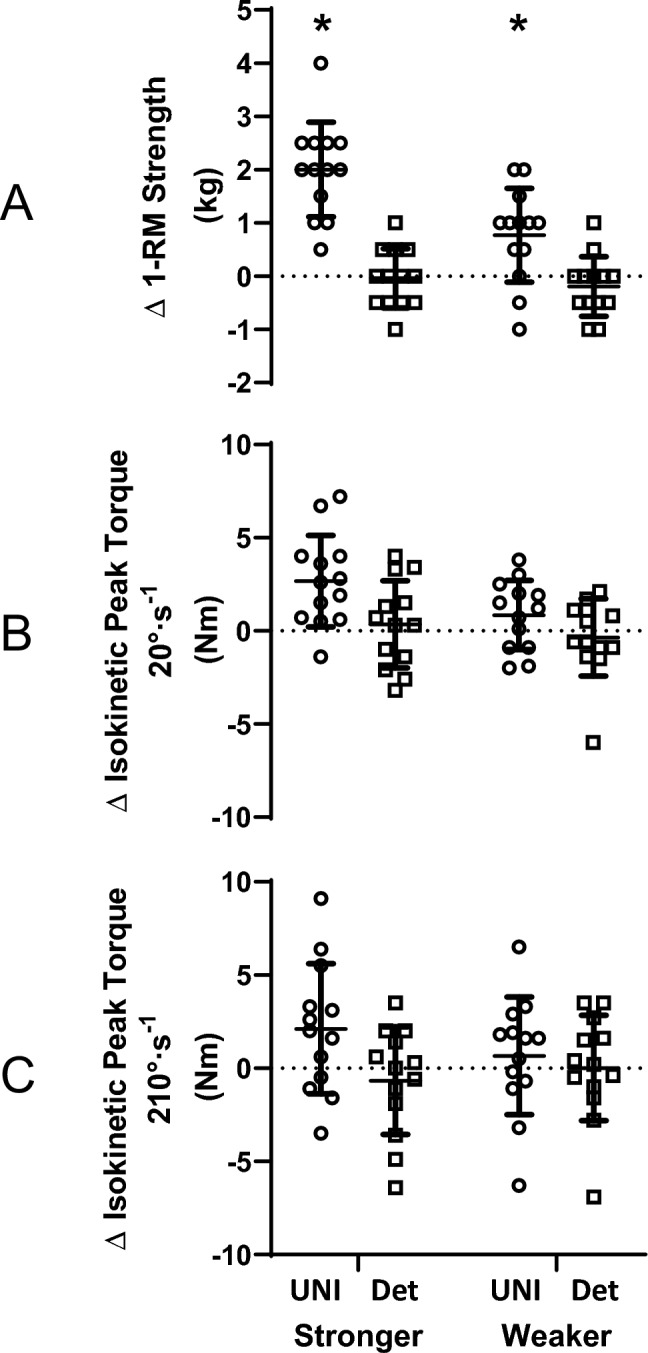
Changes in 1-RM strength (**A**) and isokinetic peak torque at 20°·s^−1^ (**B**) and 210°·s^−1^ (**C**) following either unilateral training of the stronger arm (UNI) or detraining (Det). Changes are calculated as POST values minus PRE values. Circles = UNI; squares = Detrain. *Significant change from PRE, *p* < 0.05. Symbols represent values from individual participants; horizontal lines are group means with standard deviations (SD). 1-RM strength improved in both arms following UNI but was unchanged following Detrain. Isokinetic strength was unchanged following both interventions

In UNI, pre-intervention 1-RM strength (kg) was 12.2 ± 2.7 kg for the stronger arm and 11.1 ± 2.8 kg for the weaker arm (between arms: *p* = 0.018). In Detrain, pre-intervention 1-RM strength was 12.8 ± 4.4 kg for the stronger arm and 11.8 ± 4.3 kg for the weaker arm (*p* = 0.003). Significant improvements in the trained, stronger (∆ = 2.0 ± 0.9 kg, *p* = 0.003, *g* = 0.65) and non-trained, weaker (∆ = 0.8 ± 0.9 kg, *p* = 0.022, *g* = 0.28) arms were detected following UNI, but no significant changes in the stronger (∆ = 0.0 ± 0.6 kg, *p* = 0.875, *g* = 0.02) or weaker (∆ = -0.2 ± 0.6 kg, *p* = 0.546, *g* = 0.04) arms were observed after Detrain (Fig. 2A). Interaction effects are reported in Table 2.

**Table 2:** Results summary, including mean (SD) and statistical main effects for dependent variables measured before (PRE) and after (POST) unilateral strength training of the stronger arm and detraining

	Unilateral training (UNI)				Detraining (Detrain)										
	Stronger		Weaker		Stronger		Weaker								
	PRE	POST	PRE	POST	PRE	POST	PRE	POST	Group	Arm	Time	Group × Arm	Group × Time	Arm × Time	A × T × G
1-RM strength (kg)	12.2 (2.7)	14.2 (3.1)	11.1 (2.8)	11.9 (3.0)	12.8 (4.4)	12.7 (4.4)	11.8 (4.3)	11.6 (4.3)	F_(1,24)_ = 0.007 *p* = 0.935	**F**_**(1,24)**_** = 24.476 *p* < 0.001**	**F**_**(1,24)**_** = 15.232 *p* = 0.001**	F_(1,24)_ = 0.196 *p* = 0.662	**F**_**(1,24)**_** = 20.950 *p* < 0.001**	**F**_**(1,24)**_** = 7.005 *p* = 0.014**	**F**_**(1,24)**_** = 4.672 *p* = 0.041**
Isokinetic peak torque 20°·s^−1^ (Nm)	35.5 (10.6)	38.2 (10.9)	34.4 (10.6)	35.2 (10.8)	33.6 (12.0)	34.0 (12.7)	32.8 (12.5)	32.1 (10.9)	F_(1,24)_ = 0.164 *p* = 0.689	**F**_**(1,24)**_** = 4.579 *p* = 0.043**	F_(1,24)_ = 0.812 *p* = 0.376	F_(1,24)_ = 0.363 *p* = 0.552	F_(1,24)_ = 1.527 *p* = 0.228	F_(1,24)_ = 1.593 *p* = 0.219	F_(1,24)_ = 0.039 *p* = 0.845
Isokinetic peak torque 210°·s^−1^ (Nm)	26.1 (7.2)	28.2 (7.2)	25.4 (8.0)	26.1 (8.0)	26.4 (10.9)	25.7 (9.6)	24.3 (10.0)	24.3 (8.8)	F_(1,24)_ = 0.144 *p* = 0.708	**F**_**(1,24)**_** = 10.087 *p* = 0.004**	F_(1,24)_ = 1.148 *p* = 0.295	F_(1,24)_ = 0.079 *p* = 0.781	F_(1,24)_ = 2.997 *p* = 0.096	F_(1,24)_ = 0.296 *p* = 0.591	F_(1,24)_ = 2.288 *p* = 0.143
MVIC (Nm)	50.5 (14.0)	53.5 (15.7)	48.4 (11.7)	48.7 (12.1)	49.8 (15.5)	49.6 (17.4)	47.9 (15.2)	47.7 (14.8)	F_(1,24)_ = 0.072 *p* = 0.790	**F**_**(1,24)**_** = 7.131 *p* = 0.013**	F_(1,24)_ = 1.491 *p* = 0.234	F_(1,24)_ = 0.582 *p* = 0.453	F_(1,24)_ = 2.073 *p* = 0.163	F_(1,24)_ = 2.280 *p* = 0.144	F_(1,24)_ = 2.332 *p* = 0.140
EMG_peak_ (%M_max_)	11.4 (3.1)	12.8 (5.4)	11.1 (3.7)	11.2 (3.3)	11.1 (2.9)	10.8 (3.4)	11.0 (3.6)	10.1 (4.3)	F_(1,24)_ = 0.424 *p* = 0.521	F_(1,24)_ = 0.837 *p* = 0.369	F_(1,24)_ = 0.306 *p* = 0.585	F_(1,24)_ = 0.371 *p* = 0.548	F_(1,24)_ = 2.408 *p* = 0.134	F_(1,24)_ = 4.179 *p* = 0.052	F_(1,24)_ = 1.252 *p* = 0.274
RFD_MVIC_ (%MVIC·s^−1^)	see Table 4								F_(2.23)_ = 0.492 *p* = 0.618	F_(2,23)_ = 1.704 *p* = 0.204	F_(2,23)_ = 0.227 *p* = 0.799	F_(2,23)_ = 0.515 *p* = 0.604	F_(2,23)_ = 1.078 *p* = 0.357	**F**_**(2,23)**_** = 3.915 *p* = 0.034**	F_(2,23)_ = 1.415 *p* = 0.263
EMG_40_ (%M_max_)	2.9 (1.1)	3.3 (2.1)	3.1 (2.4)	2.2 (1.3)	1.9 (1.5)	1.7 (0.8)	2.3 (1.5)	1.8 (1.2)	F_(2,23)_ = 2.583 *p* = 0.097	F_(2,23)_ = 0.064 *p* = 0.938	F_(2,23)_ = 0.761 *p* = 0.479	F_(2,23)_ = 0.952 *p* = 0.401	F_(2,23)_ = 1.039 *p* = 0.370	F_(2,23)_ = 1.288 *p* = 0.295	F_(2,23)_ = 1.525 *p* = 0.239
EMG_100_ (%M_max_)	4.5 (1.7)	5.1 (2.7)	4.4 (2.9)	4.2 (1.9)	3.1 (1.8)	3.0 (1.2)	4.1 (1.9)	2.9 (1.6)							

Results in bold highlight statistically significant effects

*A* Arm, *T* Time, *G* Group

### Elbow flexor muscle cross-sectional area (CSA_Flexor_)

**Fig. 3:**
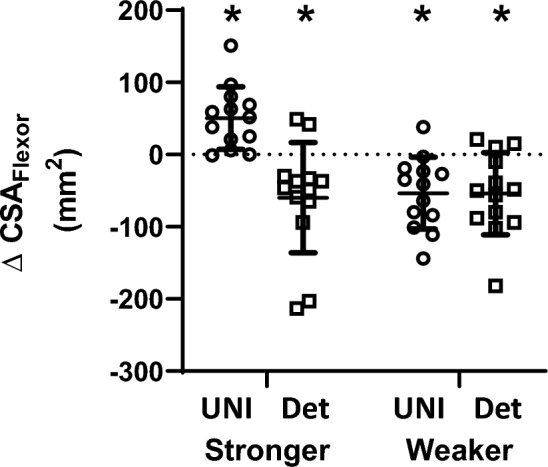
The changes in CSA_Flexor_ following either UNI or Detrain (Det). Changes are calculated as POST values minus PRE values. Circles = UNI; squares = Detrain. *Significant change from PRE, *p* < 0.05. Symbols represent values from individual participants; horizontal lines are group means with standard deviations (SD). Only the trained, stronger arm improved CSA_Flexor_ for UNI, while the non-trained, weaker arm decreased CSA_Flexor_ as did both arms for Detrain

Significant effects for CSA_Flexor_ between arms (*p* < 0.001) and time (*p* = 0.007) as well as interactions effects for group × time (*p* = 0.010), arm × time (*p* < 0.001), and arm × time × group (*p* < 0.001) were detected (Table 3). Post hoc testing revealed that CSA_Flexor_ significantly increased in the trained, stronger arm (∆ = 51 ± 43 mm^2^, *p* = 0.007, *g* = 0.12) but decreased in the non-trained, weaker arm (∆ = − 53 ± 50 mm^2^, *p* = 0.004, *g* = 0.12) following the intervention period (Fig. 3). In Detrain, there were significant decreases in both the stronger (∆ = -60 ± 77 mm^2^, *p* = 0.004, *g* = 0.12) and weaker (∆ = -54 ± 57 mm^2^, *p* = 0.004, *g* = 0.11) arms following the intervention period (Fig. 3). There were no significant correlations between changes in CSA_Flexor_ and 1-RM strength in either group (*r* < 0.496: 95% CI = − 0.134, 0.852).

**Table 3:** Results summary, including mean (SD) and statistical main effects for dependent variables measured before (PRE) and after (POST) unilateral strength training of the stronger arm and detraining

	Unilateral training (UNI)				Detraining (Detrain)										
	Stronger		Weaker		Stronger		Weaker								
	PRE	POST	PRE	POST	PRE	POST	PRE	POST	Group	Arm	Time	Group × Arm	Group × Time	Arm × Time	A × T × G
AMT (SO)	43.5 (5.9)	41.2 (4.8)	40.5 (6.1)	42.7 (7.4)	42.6 (7.2)	41.8 (7.5)	44.6 (8.1)	43.5 (7.0)	F_(1,24)_ = 0.253 *p* = 0.619	F_(1,24)_ = 0.257 *p* = 0.617	F_(1,24)_ = 0.914 *p* = 0.348	F_(1,24)_ = 1.580 *p* = 0.221	F_(1,24)_ = 0.914 *p* = 0.348	F_(1,24)_ = 2.310 *p* = 0.142	F_(1,24)_ = 3.264 *p* = 0.083
MEP 120% AMT (%M_max_)	15.7 (7.6)	14.5 (10.9)	14.7 (7.4)	15.9 (11.6)	14.3 (8.1)	16.1 (9.2)	13.1 (7.4)	13.5 (7.7)	F_(1,24)_ = 0.105 *p* = 0.748	F_(1,24)_ = 0.621 *p* = 0.438	F_(1,24)_ = 0.905 *p* = 0.351	F_(1,24)_ = 0.672 *p* = 0.420	F_(1,24)_ = 0.008 *p* = 0.931	F_(1,24)_ = 0.277 *p* = 0.603	F_(1,24)_ = 2.235 *p* = 0.148
MEP 150% AMT (%M_max_)	27.8 (13.3)	26.5 (15.3)	30.3 (17.2)	28.3 (15.7)	24.0 (13.6)	27.2 (14.4)	24.2 (10.6)	26.6 (19.4)	F_(1,24)_ = 0.340 *p* = 0.565	F_(1,24)_ = 1.066 *p* = 0.312	F_(1,24)_ = 0.060 *p* = 0.809	F_(1,24)_ = 1.010 *p* = 0.325	**F**_**(1,24)**_** = 6.105 *p* = 0.021**	F_(1,24)_ = 0.339 *p* = 0.566	F_(1,24)_ = 0.819 *p* = 0.375
cSP 120% AMT (s)	0.103 (0.025)	0.091 (0.023)	0.094 (0.022)	0.092 (0.026)	0.090 (0.019)	0.096 (0.022)	0.102 (0.026)	0.107 (0.036)	F_(1,24)_ = 0.235 *p* = 0.632	F_(1,24)_ = 0.971 *p* = 0.334	F_(1,24)_ = 0.075 *p* = 0.787	F_(1,24)_ = 4.108 *p* = 0.054	F_(1,24)_ = 2.839 *p* = 0.105	F_(1,24)_ = 0.441 *p* = 0.513	F_(1,24)_ = 0.760 *p* = 0.392
cSP 150% AMT (s)	0.130 (0.019)	0.125 (0.022)	0.124 (0.018)	0.124 (0.025)	0.119 (0.021)	0.134 (0.025)	0.129 (0.029)	0.134 (0.029)	F_(1,24)_ = 0.177 *p* = 0.677	F_(1,24)_ = 0.031 *p* = 0.861	**F**_**(1,24)**_** = 4.472 *p* = 0.045**	F_(1,24)_ = 1.756 *p* = 0.198	**F**_**(1,24)**_** = 11.845 *p* = 0.002**	F_(1,24)_ = 0.422 *p* = 0.522	F_(1,24)_ = 4.133 *p* = 0.054
pre-stimulus EMG (%M_max_)	0.6 (0.4)	0.7 (0.4)	0.6 (0.2)	0.5 (0.2)	0.5 (0.2)	0.6 (0.2)	0.5 (0.2)	0.5 (0.2)	F_(3,22)_ = 1.256 *p* = 0.314	F_(3,22)_ = 0.764 *p* = 0.526	F_(3,22)_ = 1.759 *p* = 0.184	F_(3,22)_ = 0.958 *p* = 0.430	F_(3,22)_ = 1.759 *p* = 0.402	F_(3,22)_ = 1.088 *p* = 0.375	F_(3.22)_ = 1.137 *p* = 0.356
M_max_ (mV)	16.0 (5.9)	16.1 (6.2)	16.0 (7.2)	16.4 (7.3)	17.3 (4.6)	17.4 (5.1)	16.8 (4.1)	16.8 (4.2)	F_(1,24)_ = 0.189 *p* = 0.668	F_(1,24)_ = 0.165 *p* = 0.688	F_(1,24)_ = 0.428 *p* = 0.519	F_(1,24)_ = 0.543 *p* = 0.468	F_(1,24)_ = 0.274 *p* = 0.606	F_(1,24)_ = 0.072 *p* = 0.790	F_(1,24)_ = 0.185 *p* = 0.671
CSA_Flexor_ (mm^2^)	1711 (448)	1762 (448)	1640 (461)	1586 (430)	1763 (498)	1703 (485)	1699 (504)	1645 (491)	F_(1,24)_ = 0.023 *p* = 0.881	**F**_**(1,24)**_** = 16.216 *p* < 0.001**	**F**_**(1,24)**_** = 8.640 *p* = 0.007**	F_(1,24)_ = 1.921 *p* = 0.178	**F**_**(1,24)**_** = 7.924 *p* = 0.010**	**F**_**(1,24)**_** = 19.466 *p* < 0.001**	**F**_**(1,24)**_** = 23.920 *p* < 0.001**

Results in bold highlight statistically significant effects

*A* Arm, *T* Time, *G* Group

### Isokinetic peak torque at 20°·s^−1^ and 210°·s.^−1^

Isokinetic peak torques at 20°·s^−1^ and 210°·s^−1^ (Nm) did not differ between groups (20°·s^−1^: *p* = 0.689; 210°·s^−1^: *p* = 0.708) and did not change over the experimental period (20°·s^−1^: *p* = 0.376; 210°·s^−1^: *p* = 0.295) (Fig. 2B and C). There were significant differences between arms at both contraction velocities (20°·s^−1^: *p* = 0.043; 210°·s^−1^: *p* = 0.004) (Table 2). However, no interaction effects were found at either velocity (20°·s^−1^: *p* > 0.219; 210°·s^−1^: *p* > 0.096).

### Maximal voluntary isometric contraction (MVIC) torque and peak EMG (EMG_peak_)

**Fig. 4:**
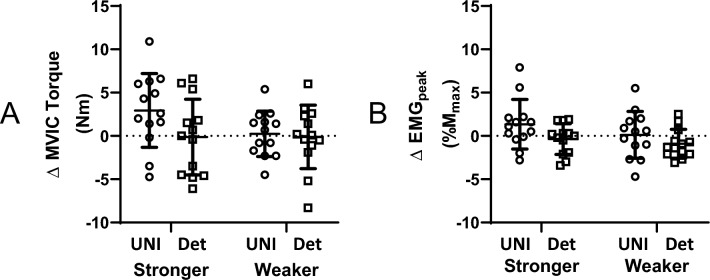
Changes in MVIC torque (**A**) and EMG_peak_ (**B**) following either UNI or Detrain (Det). Changes are calculated as POST values minus PRE values. Circles = UNI; squares = Detrain. Symbols represent values from individual participants; horizontal lines are group means with standard deviations (SD). MVIC torque and EMG_peak_ were unaltered following both interventions

MVIC torque (Nm) was not significantly different between groups (*p* = 0.790) and did not change after the experimental period (*p* = 0.234), but there were significant differences between arms (*p* = 0.013) (Table 2). However, no significant interaction effects (*p* > 0.140) were observed for MVIC torque (Fig. 4A). For EMG_peak_ (%M_max_), there were no significant group (*p* = 0.521), time (*p* = 0.585), arm (*p* = 0.369) or interaction (*p* > 0.052) effects (Table 2) (Fig. 4B). No significant correlations were detected between changes in MVIC torque and EMG_peak_ in either group (*r* < 0.369: 95% CI = -0.175, 0.740).

### Rate of force development (RFDMVIC) and EMG amplitude at 40 (EMG_40_) and 100 ms (EMG_100_) following EMG onset

RFD_MVIC_ (%MVIC·s^−1^) did not differ between arms (*p* = 0.204) or groups (*p* = 0.618) and did not change following the experimental period (*p* = 0.799). However, a significant arm × time interaction (*p* = 0.034) was detected (Table 2). Further analysis showed no changes in RFD following the intervention period (see Table 4 for RFD results). Similarly, EMG amplitude (%M_max_) measured to 40 and 100 ms did not differ between groups (*p* = 0.097) or arms (*p* = 0.938) and did not significantly change following the intervention period (*p* = 0.479) (Table 2). No significant correlations were detected between changes in RFD_MVIC_ and early EMG amplitudes in either group (*r* > -0.493: 95% CI = – 0.192, 0.834).

**Table 4:** Normalized mean (SD) rates of force development (RFD_MVIC_) in stronger and weaker arms before (PRE) and after (POST) unilateral strength training of the stronger arm and detraining. Change scores (∆) are also shown

Unilateral training			0 – 50 ms	∆	100 – 200 ms	∆
	Stronger arm (%MVIC·s^−1^)	PRE	113.8		323.9 (74.6)	
			(61.7)	41.5	311.1	− 12.9
		POST	155.3 (128.3)	(141.5)	(87.8)	(68.0)
	Weaker arm (%MVIC·s^−1^)	PRE	118.7		336.2	
			(56.1)	− 19.9	(59.6)	
		POST	98.8	(52.6)	320.2	− 16.0
			(63.7)		(73.6)	(73.1)
Detraining	Stronger arm (%MVIC·s^−1^)	PRE	132.4		310.0	
			(57.9)		(53.9)	
		POST	118.0	− 14.4	363.0	53.1
			(59.1)	(71.6)	(78.9)	(90.1)
	Weaker arm (%MVIC·s^−1^)	PRE	122.6 (46.8)		359.6 (50.5)	
		POST	99.9 (63.4)	− 22.7 (59.3)	348.1 (44.6)	− 11.5 (65.7)

∆ change, *MVIC* maximum voluntary isometric contraction

### Pre-stimulus EMG before stimulation and m-wave amplitude (M_max_)

The pre-stimulus EMG (%M_max_) did not differ between groups (*p* = 0.314) or arms (*p* = 0.526) and did not significantly change following the intervention period (*p* = 0.184) in any TMS trial (AMT, 120% and 150% of AMT). No significant group (*p* = 0.668), arm (*p* = 668) or time (*p* = 0.519) effects were detected for M_max_ (mV) (Table 3).

### Active motor threshold (AMT), motor evoked potential (MEP) amplitude at 120% and 150% of AMT, cortical silent period (cSP) duration at 120% and 150% of AMT

No significant effects for AMT (stimulator output intensity [SO]) between groups (*p* = 0.619), arms (*p* = 0.617) or time (*p* = 0.348) were detected (Table 3). Similarly, MEP amplitude at 120 and 150% of AMT (%M_max_) did not differ between groups (120%: *p* = 0.748; 150%: *p* = 0.565), arms (120%: *p* = 0.438; 150%: *p* = 0.312) or time (120%: *p* = 0.351; 150%: *p* = 0.809) (Table 3). However, a significant group × time interaction (*p* = 0.021) was detected for MEP amplitude at 150% of AMT. Further analysis revealed no changes in MEP amplitude following the experimental period. Additionally, cSP at 120% and 150% of AMT (s) did not differ between groups (120%: *p* = 0.632; 150%: *p* = 0.677) or arms (120%: *p* = 0.334; 150%: *p* = 0.861) but significant time (*p* = 0.045) and group × time interaction (*p* = 0.002) effects were detected for 150% of AMT (Table 3). Further analysis revealed that cSP significantly increased in Detrain after the 4-week detraining period (∆ = 0.010 ± 0.015 s, *p* = 0.002).

### Cross-education of strength

**Fig. 5:**
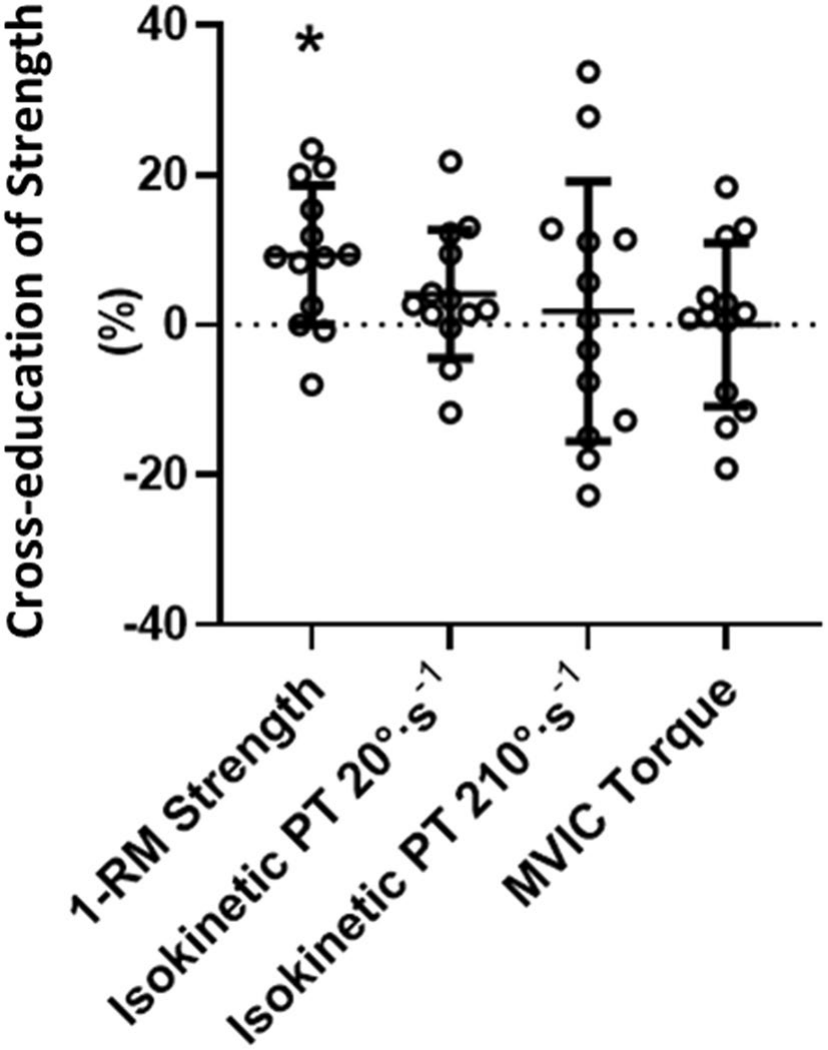
The cross-education of 1-RM strength, isokinetic peak torque at 20°·s^−1^ and 210°·s^−1^, and MVIC torque calculated using the formula original reported by Carroll and colleagues (Carroll et al. 2006). PT = peak torque. *Significantly greater than MVIC torque,* p* < 0.05. Symbols represent values from individual participants; horizontal lines are group means with standard deviations (SD). Cross-education of 1-RM strength was significantly greater than MVIC torque

1-RM strength increased 9.3 ± 9.3% more in the non-trained (weaker) arm in UNI than the weaker arm in Detrain, indicating a greater relative positive result in UNI. For isokinetic peak torque at 20°·s^−1^ and 210°·s^−1^ as well as MVIC torque, the cross-education effects were 4.1 ± 8.6%, 1.8 ± 17.4%, and 0.0 ± 11.0%, respectively (Fig. 5). For comparison between cross-education effects, post hoc testing showed that there was a significant difference between 1-RM strength and MVIC torque scores (*p* = 0.038, *g* = 0.91).

## Discussion

The novel aspect of this study was that the effect of detraining and the effectiveness of high-intensity training on cross-education of strength were assessed after the participants had completed a 4-week training period targeting the elbow flexors of both arms. Using this experimental approach, the non-trained (weaker) arm significantly increased 1-RM strength (+ 7.2%) after 4 weeks of continued unilateral strength training of the other (stronger) arm. Thus, the cross-education of strength was sufficient to further improve strength in a homologous, contralateral muscle that ceased training. A second, important, finding was that strength was maintained in the detraining group, demonstrating that the strength gains made during a short-duration (4-week), high-intensity (75% of 1-RM) training period could be retained for at least several weeks after training cessation. Muscle cross-sectional area (CSA_Flexor_) decreased in the non-trained arm following unilateral training at a similar rate to both arms following detraining, indicating that the stimulus delivered to the homologous, contralateral muscle was inadequate to retain muscle size. With regard to neurophysiological changes, the cortical silent period (cSP) increased (lengthened) in the detraining group but was maintained in both arms in the unilateral training group, indicating that the ongoing training was sufficient to maintain the level of cortical inhibition influencing descending drive to the α-motoneurons in both trained and non-trained muscles. However, as no changes in EMG amplitudes were detected and MVIC remained unchanged in both arms (and groups), the effect of cSP changes on the neural drive to the muscle and the subsequent muscular function appeared to be limited in the context of the study. In saying that, the increase in cSP following detraining may reflect the start of a process that might, over a longer time period, lead to detectable strength loss, although that possibility needs to be explicitly examined in a longer study. Overall, as there were no favorable neural or muscular changes observed in the non-trained, weaker arm following unilateral training of the stronger arm, the improvement in muscular strength may have been caused by other neural mechanisms that were not examined in the present study, such as motor learning.

Despite there being a significant existing body of research, little attention has been placed on understanding the effectiveness of cross-education to attenuate detraining effects immediately after a short (prehabilitation-type) period of strength training. The only study to examine this question reported that unilateral leg extensor training was unable to maintain muscle strength and power in the contralateral leg extensors of recreationally engaged older women (> 60 years) (de Souza Teixeira et al. 2023). These results conflict with the findings of the present study, which showed a 1-RM strength improvement in the non-trained (weaker) arm following unilateral strength training. A number of factors likely contribute to the conflicting results, including the muscle groups trained (Manca et al. 2017), but the prescription of higher relative loads in the current study is considered pivotal. With numerous studies demonstrating greater cross-education benefits training with heavier loads (Pelet and Orsatti 2021; Voskuil et al. 2023), the recommendation to train at high intensities (≥ 90% of 1-RM) is likely more important when aiming to generate a cross-education stimulus in previously trained muscles. However, more research is needed to better understand the cross-education training requirements to attenuate detraining effects in muscles that cease training. Athletes and otherwise-active populations who have become injured and clinical practitioners who prescribe pre- or post-operative exercise programs to patients stand to benefit from this knowledge.

While 1-RM strength significantly increased in both the trained and non-trained arms after the 4-week unilateral training period, isokinetic and isometric strength were not significantly increased. A likely explanation for these results is that changes were movement pattern specific in relation to muscle contraction mode (Buckner et al. 2017). In the non-trained arm, the cross-education for 1-RM strength was similar in magnitude to that previously reported after strength training (e.g., 11.9% in ref. (Manca et al. 2017)), but isokinetic and isometric strength changes were much smaller. Cross-education responses specific to contraction type have previously been reported, where unilateral eccentric training promotes contralateral eccentric strength gain, while unilateral concentric training promotes contralateral concentric strength gain (Seger and Thorstensson 2005). Based upon this line of reasoning, it is unsurprising that the isoinertial training resulted in minimal changes in MVIC and RFD_MVIC_ as both were assessed under isometric conditions. The cross-education scores calculated using the formula reported by Carroll and colleagues (2006) also indicate contraction-type specificity, with the 1-RM strength cross-education score significantly greater than the MVIC torque score (Fig. 5). In saying that, non-specific cross-education strength increases have been previously reported (Coratella et al. 2022; Martínez et al. 2021), with the contralateral elbow flexors improving MVIC following unilateral eccentric-only training (Sato et al. 2021). Although training consisting of eccentric (and concentric) muscle actions was prescribed in the present study, it is acknowledged that unilateral eccentric-focused training promotes greater cross-education benefits (Voskuil et al. 2023). In the trained arm, a significant increase in 1-RM strength (+ 16.4%) but no significant changes in isokinetic torque or MVIC torque was also detected (see Fig. 2 and 4A). Thus, heavy-load isoinertial training resulted in improvements in isoinertial strength of the trained and non-trained arm without influencing isokinetic and isometric strength. Importantly, however, isokinetic peak torques at 20°·s^−1^ and 210°·s^−1^ did significantly improve following the 4 weeks of isoinertial training that preceded the current study (Rowe et al. 2023), suggesting perhaps that some non-specific improvement can occur in the first weeks of training but that ongoing improvements are less likely. Some support for this is provided by Blazevich and colleagues, who found that participants who had less ability to produce force rapidly were able to improve RFD following slow-speed strength training, but those who had above-median RFD ability did not (Blazevich et al. 2008). Also, the participants who responded to training largely improved RFD within the first five weeks, with little change occurring once the participants reached the same RFD capacity as their counterparts. Collectively, these findings suggest that training specificity may become more important as training progresses.

An important finding of the present study was that there were no significant strength changes after 4 weeks of detraining of both arms. Previously, decreases in maximal strength have been observed within 3–5 weeks of detraining (McMaster et al. 2013), although other studies indicate that maximal strength can be preserved for several months (Issurin and Lustig 2004; Counsilman and Counsilman 1991). As upper body muscles are less likely to receive a stimulus during habitual activities to attenuate the effects of detraining, we expected that the elbow flexors would be susceptible to strength loss. However, at least one study reported that maximal elbow extensor strength can be preserved for 12 months following 12 weeks of isokinetic training (Popadic Gacesa, Dusko, Grujic 2011). In contrast, as RFD has been reported to decline to baseline within three weeks of the cessation of explosive strength training (Kobayashi et al. 2013), it was expected that RFD_MVIC_ would show signs of decrement. However, as neither RFD nor the EMG measured in the early period after muscle activation were detectibly improved after 4 weeks of training prior to the study (Rowe et al. 2023), there was little scope for RFD and EMG loss during subsequent detraining.

Although unilateral training of the stronger arm improves 1-RM strength in the non-trained, weaker arm, the improvement did not result from hypertrophic adaptations as muscle size decreased in the weaker arm at a similar rate to both arms in the detraining group. Consequently, the immediate training history of the muscle likely impacts the effectiveness of cross-education to influence local muscle properties, given that other research has demonstrated that muscle size may be preserved in an immobilized limb following unilateral strength training (Farthing, Krentz, Magnus 2009; Pearce et al. 2013). Given the results of the present study, the stimulus appears to be inadequate to prevent muscle atrophy in muscles that were previously trained. Although CSA_Flexor_ decreased in the exercise-restricted limbs of both groups, it increased in the exercised limb of the unilateral training group (+ 2.5%). This significant increase was smaller in magnitude than the change reported in our previous study (+ 6.6%) (Rowe et al. 2023), indicating that the rate of muscle size increase slows with continued training in the elbow flexors. Also, there was no significant correlation between the changes in CSA_Flexor_ and 1-RM strength which has been detected previously (Rowe et al. 2023), indicating that hypertrophic contribution to force production in the elbow flexors may decrease with continued training.

MEP amplitude changes were not detected after the training, despite previous reports of MEP increases in an isometric contraction after training (Weier et al. 2012; Goodwill et al. 2012). Again, this lack of effect may reflect exercise mode specificity, such that MEPs evoked during low-torque isometric tests may not be altered by the isoinertial training. Recently, MEPs evoked during isokinetic lengthening or shortening contractions after either lengthening or shortening training were found to show increases only during lengthening contractions after lengthening training (Tallent et al. 2017). Therefore, changes may be more detectable when the testing and training methods are contraction type compatible. Similarly, the lack of muscle activation change is consistent with the previous finding that changes in isometric peak torque in response to isoinertial training were not related to changes in muscle activity (Trezise and Blazevich 2019).

A novel finding of the present study was that cSP duration was maintained for the unilateral training group but lengthened in the detraining group. cSP reflects a suspension of descending drive from the motor cortex and subsequent disfacilitation of the motoneurons. It is primarily caused by intracortical inhibition mediated through γ-aminobutyric acid B (GABA_b_) receptors (Werhahn et al. 1999). Therefore, the present findings suggest that detraining was associated with an increase in intracortical inhibition, but that this was prevented by continued unilateral training. Increased intracortical inhibition might be expected to influence descending drive to the motoneurons (and, thus, the muscle); however, no changes in muscle activity or MVIC strength were detected in either arm of either group. A possible explanation for these conflicting results is that there may be little association between cortical responses and muscle function after the initial stages of training. For example, Griffin and Cafarelli demonstrated that MEP amplitude and MVIC torque both significantly increased after two weeks of strength training, but MEP amplitude remained unaltered, whilst MVIC torque continued to increase after a further two weeks of training (Griffin and Cafarelli 2007). A similar outcome was observed by Mason and colleagues (2020), who found a decrease in cSP after one week of strength training that triggered a 1-RM strength improvement, but cSP was numerically increasing when 1-RM strength continued to improve following a second week of training. Tallent and colleagues also reported that maximal isokinetic strength and muscle activation were unchanged following two weeks of detraining, whilst MEP amplitude significantly decreased (Tallent et al. 2017). In the current study, cross-education of 1-RM strength occurred without cortical changes related to either the trained or non-trained arm. Thus, although several studies show that contralateral and ipsilateral cortical changes are important for strength adaptations in trained (Mason et al. 2020; Coombs et al. 2016; Latella, Kidgell, Pearce 2012) and non-trained muscles (Leung et al. 2018; Goodwill, Pearce, Kidgell 2012), the likelihood that further cortical changes facilitate strength benefits in previously trained muscles may be limited.

While unilateral strength training improves activation in homologous, contralateral muscles (Green and Gabriel 2018; Lee, Gandevia, Carroll 2009), alterations in motor strategy (learning) might at least partly explain the effect (Ruddy and Carson 2013); the 'bilateral access' hypothesis is one theoretical model of this phenomenon (Ruddy and Carson 2013; Lee et al. 2010). According to this hypothesis, the repeated performance of specific motor skills generates motor engrams or stored memories. These motor engrams are established in brain centers that are not only accessible to both hemispheric motor networks or are established in the trained hemisphere but also accessible to the contralateral motor networks due to callosal connections (Ruddy and Carson 2013). Recently, researchers have tried to determine the task dependence of cross-education by comparing TMS and performance responses between strength and skill training interventions (Leung et al. 2018). As MEP amplitudes increase and short-interval intracortical inhibition decrease similarly following paced strength training (using a metronome) and skill training but not after self-paced strength training, it was speculated that paced contractions under load provides a similar motor learning stimulus to skill exercises (Leung et al. 2018). In the present study, no MEP changes were observed even though the training contractions were paced with a metronome. A possible reason why motor learning-related MEP changes were not detected is that the participants had trained their elbow flexors for four weeks before undertaking this study (Rowe et al. 2023). As the participants had already performed ~ 300 paced preacher curls with both arms, the chance of further motor learning might have been minimal. However, a learning opportunity in the present study was that the training intensity was greater (90% of 1-RM as opposed to 75% of 1-RM), with recent evidence demonstrating that inhibitory cortical responses after a single heavy-load (80% of 1-RM) strength exercise session resemble those after skill practice rather than those after low-load (20% of 1-RM) strength exercise (Mason et al. 2019). These findings suggest that strength training (at least at heavy loads) might be considered a form of motor learning. Therefore, despite the participants’ familiarity with the training exercise, the unfamiliar extra load that more closely resembles maximal strength testing may have prompted additional motor learning that resulted in 1-RM strength improvement in the contralateral elbow flexors. This theory is supported by previous research showing enlarged regions of activation in the contralateral sensorimotor cortex and left temporal lobe during muscle contractions with the untrained left arm following maximal strength training (Farthing et al. 2011). These results suggest that adaptations in the sensorimotor cortex and temporal lobe may contribute to the cross-education of strength, which is consistent with previous reports of motor learning (Grafton et al. 1995) and semantic memory of movement (Martin 2001; Staines et al. 2002). Future work on the cross-education of strength should examine mechanisms associated with motor learning to explain these findings.

Some key points need to be discussed regarding the implications of the results of the current study. Previous evidence indicates that maximal strength, muscle size, and corticospinal excitability can be retained in an immobilized limb due to the cross-education of strength. This is important as the adverse effects of limb immobilization can cause significant strength loss and neuromuscular impairment, increasing the recovery time needed to restore function to pre-trauma levels. Importantly, this study confirmed that the cross-education of strength can improve maximal strength in a previously trained muscle; however, it did not attenuate muscle loss. Pertinent to athletic and otherwise-active populations as well as clinical practitioners who prescribe pre- or post-operative exercise programs to patients, the cross-education stimulus generated with high-intensity training can prevent strength loss in a strength-trained limb subjected to exercise restriction. However, direct training will be required to redevelop muscle size following disuse interventions. Strength adaptations were also retained for muscles that did not receive a training stimulus following a short (prehabilitation-type) period of strength training. Thus, the short-term development of strength, specifically in the elbow flexors, can be maintained for several weeks during training cessation. Together, these findings provide support to the implementation of pre/rehabilitation protocols to aid strength recovery following pre-planned surgeries.

The findings of the current study must be considered in view of some limitations. First, although a comprehensive experimental protocol was implemented, consisting of tests examining neurophysiological and muscular mechanisms to better understand the cross-education of strength, the findings have raised more questions than answers. Other researchers have since highlighted that alternative testing techniques show promise in the study of the mechanisms of cross-education, including functional magnetic resonance imaging, and paired pulse TMS to assess short-interval intracortical inhibition and interhemispheric inhibition (Manca et al. 2021). Future research should utilize these testing techniques to examine the mechanisms of the cross-education of strength, especially in individuals with previous training experience. Second, the moderate sample size will influence the statistical power of the study, potentially increasing Type I and II error risks and making it more difficult to control for confounding factors that can introduce bias. However, a post hoc power analysis shows the sample size was sufficient to identify differences in 1-RM improvement in the trained and non-trained arms of the training group compared to the arms of the detraining group (group x time interaction: 1 – β err prob = 0.99), and the sample size is consistent with other repeated-measures studies that have implemented similar protocols used to investigate strength training adaptations, including the cross-education of strength (Leung et al. 2018; Coombs et al. 2016). The data dispersion in some testing outcomes may also raise concerns about the uncertainty in the parameter estimates, but it is believed the mixed study sample largely contributes to the data distribution in those measures which improves the generalizability of the findings. Ultimately, we report the means and standard deviations of the dependent variables at each testing time point and provide informative figures of the key findings. This information should allow readers to make their own judgment on the outcomes. Third, the lack of experimental concealment is a challenge in most strength training studies, but adherence also becomes a greater challenge when instructing participants to cease exercise. While participant retention was high in the study, adherence to the instruction to stop exercise or limit the performance of incidental high-intensity efforts was not examined. Future studies investigating detraining effects should seek to quantify the physical activity levels of participants, while they are away from the experiment. Nonetheless, the consistent reduction in CSA_Flexor_ in the untrained arms of both groups provides confidence the participants were compliant. Finally, the evaluation of testing measurement error was not a focus of the study, and thus, details regarding the minimal detectable change cannot be provided from the study data. However, participants in the current study were highly familiar with the testing techniques as they had previously undertaken the same testing procedures in another study (Rowe et al. 2023). Moreover, evidence from other research into 1-RM strength testing of the elbow flexors in younger men and women highlights its low variability (Grosicki et al. 2014). Given the short training period, the mixed study sample, and the fact that larger strength gains are less likely in individuals with previous training experience, the strength improvement is considered a positive meaningful change of muscular function and not measurement error.

In conclusion, the present results demonstrate that 1-RM strength can improve in the non-trained, weaker arm in response to four weeks of unilateral strength training by the stronger arm, even when a short (prehabilitation-type) period of training was previously provided to both arms; the most novel aspect of this finding is that the improvement was generated in previously trained muscles using high-intensity loads (90% of 1-RM). However, muscle size decreased and there was a lack of detectable changes in many tests of neural function, so the precise mechanism/s underpinning the strength effects cannot be determined from the present data. Untested neural mechanisms, possibly related to motor learning, may have contributed to the improvement in muscular strength in the contralateral arm. Lastly, the lack of strength change in the detraining group indicates that short-term development of strength, specifically in the elbow flexors, can be maintained for several weeks during training cessation. From a practical perspective, the lack of strength loss could be of great clinical use, since it seems that significant strength enhancement in response to only a short period of training can be maintained for several weeks after training cessation. This might be important as a prehabilitation tool and should be tested in a clinical population in the future.

## Supplementary Information

Below is the link to the electronic supplementary material.

Supplementary file1 (DOCX 16 kb)

## Data Availability

The data that support the findings of this study are available from the corresponding author upon reasonable request.
