# Supplementary material for: Can the cross-education of strength attenuate the impact of detraining after a period of strength training? A quasi-randomized trial
Source: Eur J Appl Physiol. 2024 May 29;124(10):1–16. doi: 10.1007/s00421-024-05509-z (PMC11467040; doi:10.1007/s00421-024-05509-z)
Supplement: Supplementary file 1 — Supplementary file1 (DOCX 16 kb) [file 421_2024_5509_MOESM1_ESM.docx]

**Supplementary material 1.** Relationships between changes in specific dependent variables in both arms following unilateral strength training by the stronger arm or detraining.

| **Unilateral training** |  |  | **Stronger arm** |  | **Weaker arm** |
| --- | --- | --- | --- | --- | --- |
|  |  |  |  |  |  |
|  | 1-RM vs. |  | CSA_Flexor_ |  | CSA_Flexor_ |
|  |  |  | -0.067 (-0.539, 0.412) |  | 0.496 (-0.134, 0.852) |
|  |  |  |  |  |  |
|  | MVIC vs. |  | EMG_peak_ |  | EMG_peak_ |
|  |  |  | 0.369 (-0.175, 0.740) |  | 0.154 (-0.423, 0.739) |
|  |  |  |  |  |  |
|  | RFD_50/MVIC_ vs. |  | EMG_40_ |  | EMG_40_ |
|  |  |  | -0.202 (-0.750, 0.566) |  | -0.110 (-0.562, 0.451) |
|  |  |  |  |  |  |
|  | RFD_100-200/MVIC_ vs. |  | EMG_100_ |  | EMG_100_ |
|  |  |  | 0.073 (-0.347, 0.512) |  | 0.396 (-0.264, 0.828) |
|  |  |  |  |  |  |
| **Detraining** |  |  | **Stronger arm** |  | **Weaker arm** |
|  |  |  |  |  |  |
|  | 1-RM vs. |  | CSA_Flexor_ |  | CSA_Flexor_ |
|  |  |  | 0.260 (-0.117, 0.640) |  | 0.103 (-0.422, 0.507) |
|  |  |  |  |  |  |
|  | MVIC vs. |  | EMG_peak_ |  | EMG_peak_ |
|  |  |  | 0.348 (-0.223, 0.857) |  | -0.091 (-0.739, 0.597) |
|  |  |  |  |  |  |
|  | RFD_50/MVIC_ vs. |  | EMG_40_ |  | EMG_40_ |
|  |  |  | -0.493 (-0.192, 0.834) |  | 0.137 (-0.294, 0.629) |
|  |  |  |  |  |  |
|  | RFD_100-200/MVIC_ vs. |  | EMG_100_ |  | EMG_100_ |
|  |  |  | 0.312 (-0.443, 0.730) |  | -0.174 (-0.636, 0.319) |

No significant relationships were observed; 95% confidence interval (CI) crossed 0.00 in all cases
